# Association of quadriceps muscle, gluteal muscle, and femoral bone marrow composition using chemical shift encoding-based water-fat MRI: a preliminary study in healthy young volunteers

**DOI:** 10.1186/s41747-020-00162-5

**Published:** 2020-06-10

**Authors:** Michael Dieckmeyer, Florian Zoffl, Lioba Grundl, Stephanie Inhuber, Sarah Schlaeger, Egon Burian, Claus Zimmer, Jan S. Kirschke, Dimitrios C. Karampinos, Thomas Baum, Nico Sollmann

**Affiliations:** 1 Department of Diagnostic and Interventional Neuroradiology, Klinikum rechts der Isar, Technische Universität München, Ismaninger Str. 22, 81675 Munich, Germany; 2 Department of Sport and Health Sciences, Technische Universität München, Georg-Brauchle-Ring 60/62, 80992 Munich, Germany; 3 Department of Diagnostic and Interventional Radiology, Klinikum rechts der Isar, Technische Universität München, Ismaninger Str. 22, 81675 Munich, Germany; 4 TUM-Neuroimaging Center, Klinikum rechts der Isar, Technische Universität München, Munich, Germany

**Keywords:** Bone marrow, Femur, Healthy volunteers, Magnetic resonance imaging, Muscles

## Abstract

**Background:**

We investigated the composition of the gluteal (gluteus maximus, medius, and minimus) and quadriceps (rectus femoris, vastus lateralis, medialis, and intermedius) muscle groups and its associations with femoral bone marrow using chemical shift encoding-based water-fat magnetic resonance imaging (CSE-MRI) to improve our understanding of muscle-bone interaction.

**Methods:**

Thirty healthy volunteers (15 males, aged 30.5 ± 4.9 years [mean ± standard deviation]; 15 females, aged 29.9 ± 7.1 years) were recruited. A six-echo three-dimensional spoiled gradient-echo sequence was used for 3-T CSE-MRI at the thigh and hip region. The proton density fat fraction (PDFF) of the gluteal and quadriceps muscle groups as well as of the femoral head, neck, and greater trochanter bone marrow were extracted and averaged over both sides.

**Results:**

PDFF values of all analysed bone marrow compartments were significantly higher in men than in women (*p* ≤ 0.047). PDFF values of the analysed muscles showed no significant difference between men and women (*p* ≥ 0.707). After adjusting for age and body mass index, moderate significant correlations of PDFF values were observed between the gluteal and quadriceps muscle groups (*r* = 0.670) and between femoral subregions (from *r* = 0.613 to *r* = 0.655). Regarding muscle-bone interactions, only the PDFF of the quadriceps muscle and greater trochanter bone marrow showed a significant correlation (*r* = 0.375).

**Conclusions:**

The composition of the muscle and bone marrow compartments at the thigh and hip region in young, healthy subjects seems to be quite distinct, without evidence for a strong muscle-bone interaction.

## Key points

- Quantitative magnetic resonance imaging-based assessment of thigh muscle and femoral bone marrow composition is feasible with good inter-reader reproducibility.
- Only moderate associations between compositions of quadriceps muscles, gluteus muscles, and different femoral bone marrow compartments were revealed.
- Regarding fat content, there is no evidence for a strong muscle-bone interaction at the thigh and hip region in healthy young subjects.

## Background

The potential relationship between the composition of musculature and bone marrow compartments of the thigh and hip region is of profound interest as it reflects a closely interacting functional unit that is the foundation of our mobility [1]. Initially, x-ray-based imaging modalities have been used for the quantitative analysis of tissue composition and volume. Dual-energy x-ray absorptiometry enables the assessment of bone mineral density as well as lean and adipose tissue mass [2]. Furthermore, assessment of muscle composition has been extensively performed by indirect density-based quantification of inter-muscular adipose tissue using computed tomography, which allows to simultaneously derive measurements of muscle volume [3]. More recently, magnetic resonance imaging (MRI) has evolved as a technique to perform quantitative analysis of different tissues of the human body. In particular, chemical shift encoding-based water-fat magnetic resonance imaging (CSE-MRI) and magnetic resonance spectroscopy can measure the proton density fat fraction (PDFF) in skeletal muscle [4], bone marrow [5], the liver [6–8], and other tissues [9]. The spatial resolution of CSE-MRI provides the opportunity to analyse multiple compartments from the same dataset, thereby enabling the efficient PDFF assessment of muscles and bone marrow of the thigh and hip region.

In general, water-fat composition of both muscle and bone marrow has been investigated in previous studies and linked to a variety of pathological conditions including musculoskeletal disorders, *e.g*., osteoporosis and sarcopenia [10–12]; neuromuscular disorders, *e.g*., Duchenne muscular dystrophy [13–17]; and metabolic disorders, *e.g*., type 2 diabetes mellitus [18]. At the lumbar spine, the association between muscular and bone marrow compartments has been previously investigated in healthy subjects [19]. Using CSE-MRI, a significant correlation between the PDFF of paraspinal muscles and vertebral bone marrow in postmenopausal women was revealed [19]. Additionally, in the context of osteoporosis, a CSE-MRI-based analysis of fat content and composition of different bone marrow subregions of the femur has been performed and demonstrated significant differences between pre- and postmenopausal women [20]. In a study with patients suffering from anorexia nervosa, fat content and composition of different bone marrow subregions of the thigh and hip region were quantified by means of magnetic resonance spectroscopy and compared to body fat percentage and bone mineral density [21]. The results suggested that the relationships between bone marrow composition and body fat content are quite complex and exhibit regional differences [21]. Preliminary studies also showed that an alteration of muscle composition, in particular increased fatty infiltration, entails negative implications for muscle strength and function at the thigh [22, 23].

However, the influence of age and body mass index (BMI) on water-fat composition of the muscular and osseous components of the thigh and hip region as well as the potential relationships between compartments using PDFF measurements have not been investigated to date. Considering the close anatomical and functional interactions of the proximal leg muscles and the femur, analysing the association of these measurements could provide novel insights into the physiology of the musculoskeletal system. Against this background, the aim of this retrospective study is to systematically investigate the association between the PDFF of the gluteus and quadriceps femoris muscle groups as well as the femoral head, neck, and greater trochanter bone marrow using CSE-MRI in healthy adults. Such association could potentially suggest a common underlying cause for changes in muscle and bone marrow composition and thereby reveal a connection between impaired muscle function and bone quality in the thigh and hip region.

## Methods

### Subjects

Thirty healthy volunteers were recruited for this retrospective study. The same cohort has been under investigation in a previous study, but with different purposes [24]. The inclusion criteria were (i) age between 20 and 40 years and (ii) BMI between 20 and 33 kg/m^2^. Exclusion criteria were (i) prevalent or history of metabolic disorders, neuromuscular diseases, spine or thigh trauma; (ii) body conditions related to disbalance and/or morphological asymmetry at the level of the hip (such as scoliosis, advanced hip arthrosis); and (iii) general MRI contraindications. No subject reported any major physical conditions limiting mobility, and all subjects were considered having a normally active lifestyle. All subjects were right-footed.

Written informed consent was obtained from all subjects enrolled in this study. The study protocol was in accordance with the Declaration of Helsinki and its later amendments and was approved by the local institutional review board. The time between data acquisition of the first and last subject of the study was 4.5 months, and the interval for study inclusion was from April 2019 to August 2019.

### Magnetic resonance imaging

All subjects underwent 3-T MRI (Ingenia, Philips Healthcare, Best, The Netherlands; bore diameter 70 cm, maximum field of view 55 cm) in a supine position using the built-in-the-table posterior and an anterior coil (32 channels, 60 cm coverage in feet-head direction, multi-array surface coils).

The imaging protocol, a standard protocol for quantitative MRI of the hip and thigh region at our institution, comprised an axial six-echo three-dimensional spoiled gradient-echo sequence for chemical shift encoding-based water-fat separation at the bilateral thigh and hip region. Sequence parameters were set as follows: repetition time 6.4 ms, echo time 1.1 ms, *Δ* echo time 0.8 ms, field of view 220 × 401 × 252 mm^3^, acquisition matrix 68 × 150, voxel size 3.2 × 2.0 × 4.0 mm^3^, frequency encoding direction left-to-right, no parallel imaging, and scan time 1:25 min:s per stack. Images were acquired in two stacks to cover the volume of the upper endplate of L4 down to the mid-thigh region. The six echoes were acquired in a single repetition time using non-flyback (bipolar) read-out gradients. A flip angle of 3° was used to minimise T1 bias effects [25, 26].

### Muscle and femur compartment segmentation and PDFF extraction

**Fig. 1:**
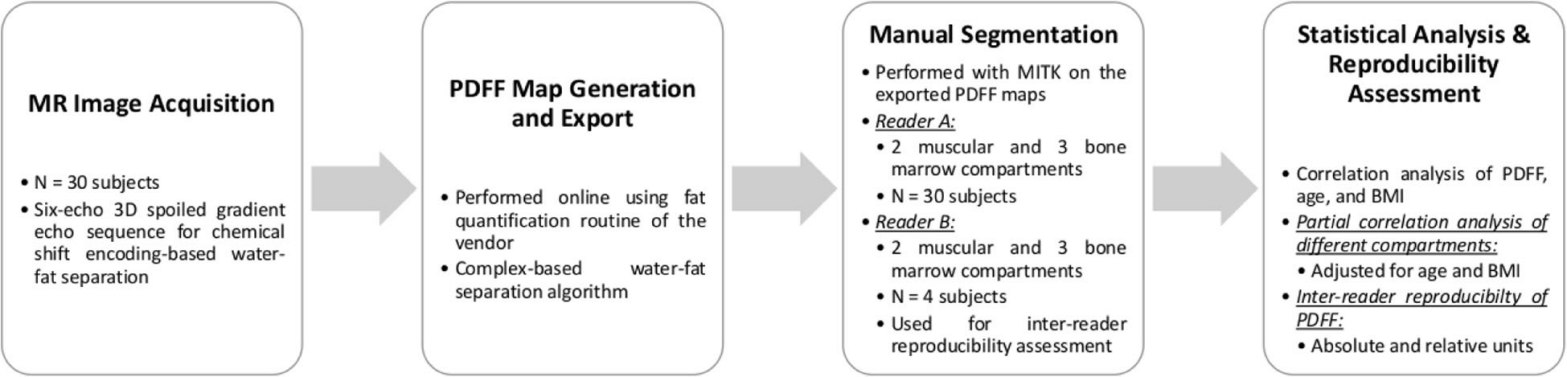
Overview of the image acquisition, postprocessing workflow, and image data analyses. *BMI,* Body mass index; *PDFF*, Proton density fat fraction

The gradient-echo imaging data were processed online using the fat quantification routine of the MRI vendor (Philips Healthcare, Best, The Netherlands). PDFF maps were generated using a complex-based water-fat separation algorithm that accounts for known confounding factors including a single T2* correction, phase error correction, and consideration of the spectral complexity of lipids using the multi-peak fat spectrum model of Ren et al. [27]. Segmentation was performed by a medical doctor (F.Z.), supervised by a radiologist (T.B.) with 9 years of experience, using the free open-source software Medical Imaging Interaction Toolkit (MITK; developed by the Division of Medical and Biological Informatics, German Cancer Research Center, Heidelberg, Germany; www.mitk.org; Fig. 1).

**Fig. 2:**
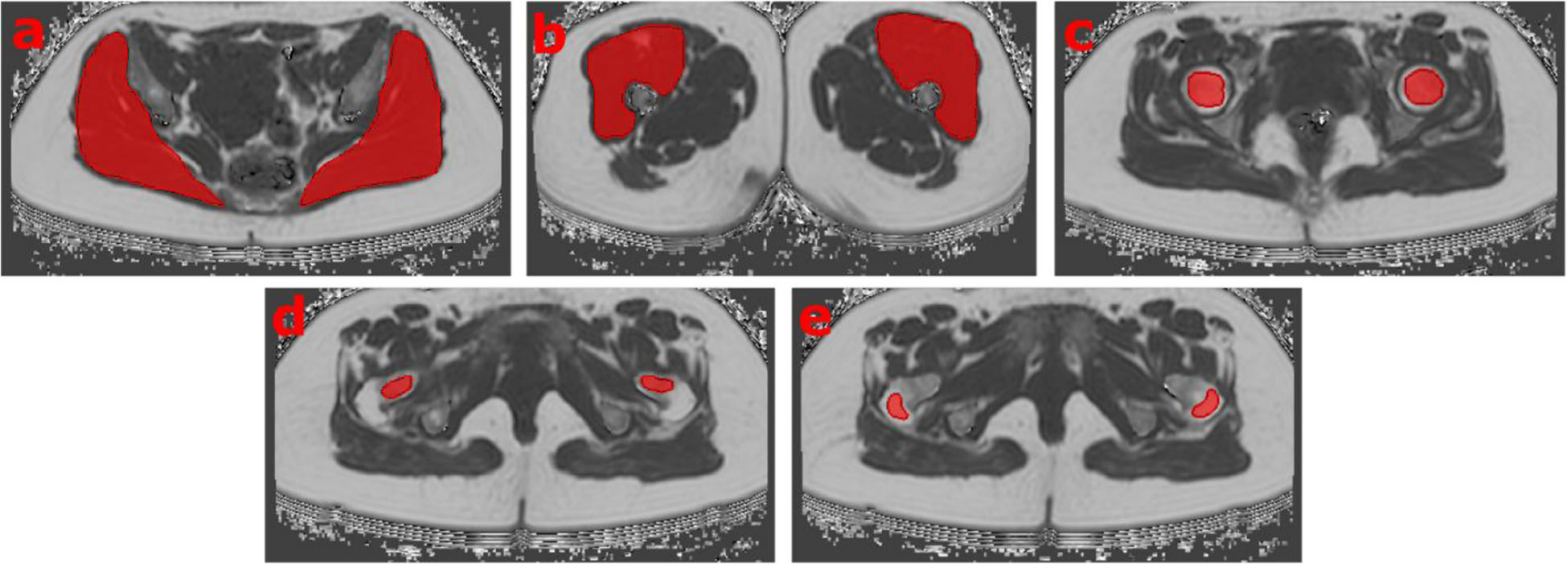
Representative segmentations (red areas) of the five muscular and osseous compartments, prescribed on axial proton density fat fraction maps of a 39-year-old female subject (body mass index 25.7 kg/m^2^): gluteal (**a**), quadriceps (**b**), femoral head (**c**), femoral neck (**d**), and greater trochanter (**e**)

The gluteus and quadriceps femoris muscle groups as well as three subregions of the femur (head, neck, and greater trochanter) were manually segmented on both sides in the PDFF maps (Figs. 1 and 2). No supportive semiautomatic or automatic segmentation techniques (such as thresholding or region growing) were used. Segmentation of the quadriceps muscle included all four compartments (rectus femoris, vastus lateralis, vastus medialis, and vastus intermedius) and was performed in five consecutive axial slices, starting ten axial slices caudal of the small trochanter. Segmentation of the gluteal muscle included all three compartments (gluteus maximus, gluteus medius, and gluteus minimus) and was performed in five consecutive axial slices, with the centre slice being located at the level of the maximum diameter of the piriformis muscle. Segmentation of the femur subregions was performed with sufficient distance to the cortical bone in order to avoid the inclusion of tissue other than bone marrow. The PDFF [%] was extracted and averaged over both sides, which was achieved separately for the muscle (PDFF_gluteus_ and PDFF_quadriceps_) and femoral bone marrow compartments (PDFF_femoral head_, PDFF_femoral neck_, and PDFF_femoral greater trochanter_).

### Reproducibility of PDFF measurements

Four randomly selected subjects (two males and two females) from the study population were used to determine the inter-reader reproducibility of the PDFF measurements. All muscle and bone marrow segmentations as described above were performed independently in those four subjects by a second reader (L.G., 4 years of experience in image segmentation and analysis).

### Statistical analysis

Statistical analyses were performed with SPSS (SPSS Inc., Chicago, IL, USA). All statistical tests were conducted using a two-sided level of significance equal to *α* = 0.05.

The Kolmogorov-Smirnov test was used to test for normal distribution of the measured parameters. Mean and standard deviation (SD) were calculated for age and PDFF of the three bone marrow and two muscle compartments (parametric data distribution for age and PDFF). Median and interquartile range (IQR) were calculated for BMI (non-parametric data distribution for BMI). Values were compared between males and females using Mann-Whitney *U* tests or unpaired *t* tests, depending on non-parametric *versus* parametric data distribution. Correlations of PDFF, age, and BMI were analysed using Spearman’s Rho. Furthermore, partial correlation analyses between PDFF values of different compartments were performed adjusting for BMI and age.

The inter-reader reproducibility error for the four subjects analysed by the two readers was expressed as the root mean square of the absolute precision error (absolute units) and root mean square of the relative precision error (expressed as coefficient of variation, relative units) according to Gluer et al. [28].

## Results

### Study population

The assumption of a normal distribution had to be rejected for BMI. All other analysed parameters were assumed to be normally distributed. There was no statistically significant difference between male and female subjects regarding age. The women’s age was 29.9 ± 7.1 years (mean ± SD), ranging from 21 to 42 years; the men’s age was 30.5 ± 4.9 years, ranging from 23 to 41 years (*p* = 0.790). The women’s median BMI was 26.1 kg/m^2^ (IQR 2.8 kg/m^2^, range 23.7–28.4 kg/m^2^); the men’s median BMI was 26.3 kg/m^2^ (IQR 5.5 kg/m^2^, range 24.3–32.5 kg/m^2^) (*p* = 0.300) (Table 1).

**Table 1:** Study population characteristics

	Males (*n* = 15)	Females (*n* = 15)	*p* value
Age (years)	30.47 ± 4.90	29.87 ± 7.13	0.790
Body mass index (kg/m^2^)	26.25, 5.50	26.13, 2.77	0.300
PDFF_gluteus_ (%)	5.25 ± 1.73	5.49 ± 1.83	0.707
PDFF_quadriceps_ (%)	2.53 ± 1.00	2.65 ± 1.02	0.714
PDFF_femoral head_ (%)	86.29 ± 8.25	83.69 ± 6.07	0.047*
PDFF_femoral neck_ (%)	73.31 ± 10.59	62.87 ± 8.70	0.006*
PDFF_femoral greater trochanter_ (%)	92.13 ± 2.20	90.26 ± 2.39	0.034*

Data are given as mean ± standard deviation for age and PDFF, and as median and interquartile range for body mass index. *PDFF,* Proton density fat fraction

*Statistical significance (*p* < 0.05)

### PDFF measurements

**Fig. 3:**
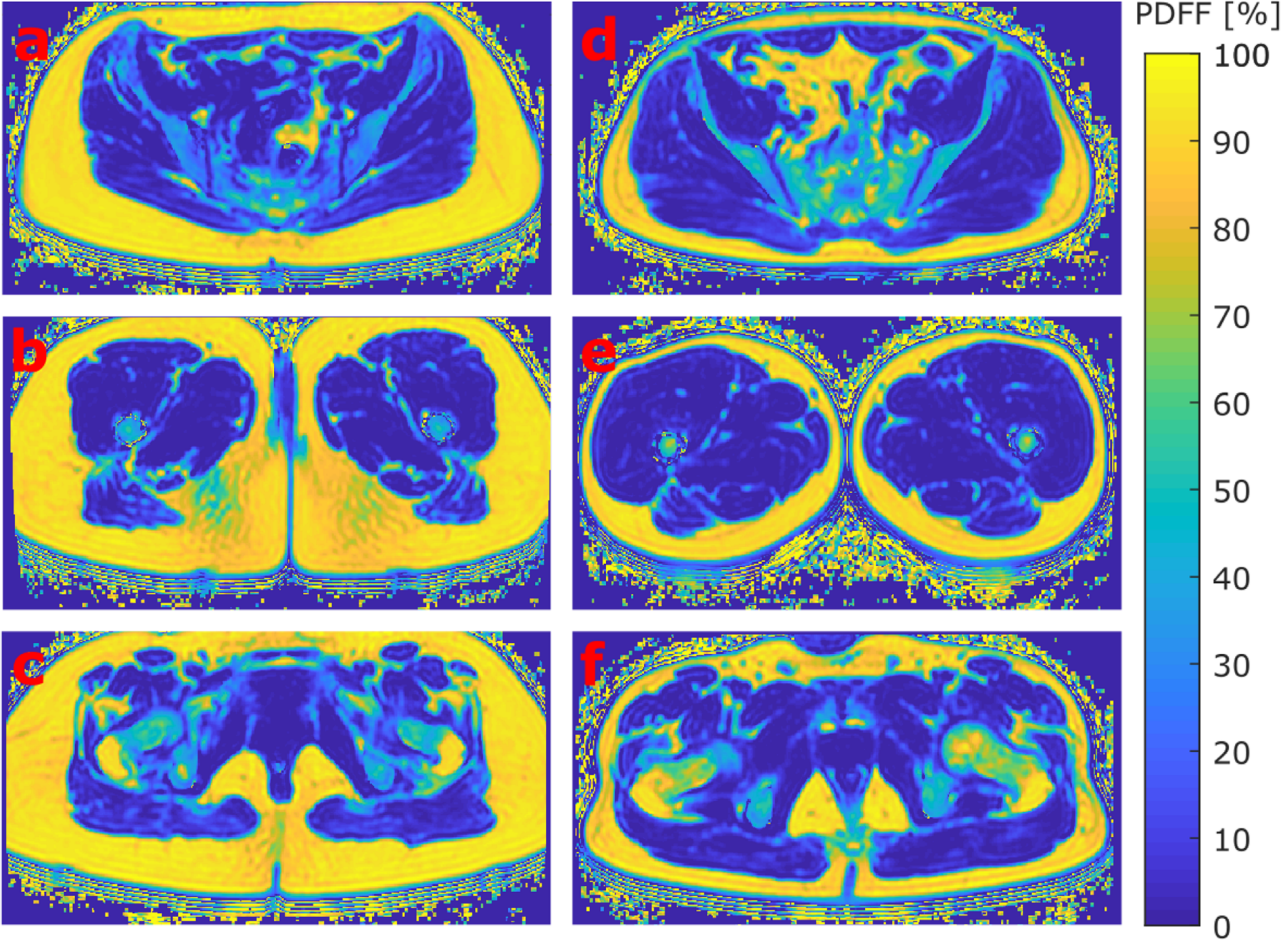
Representative colour-coded axial PDFF maps of the gluteal (**a**, **d**), quadriceps (**b**, **e**), and femoral neck (**c**, **f**) regions of a 39-year-old female subject (BMI 25.7 kg/m^2^, left column **a**–**c**) and a 41-year-old male subject (BMI 25.8 kg/m^2^, right column **d**–**f**), respectively. *BMI*, Body mass index; *PDFF*, Proton density fat fraction

Mean and SD of the PDFF of the five segmented compartments are shown in Table 1. In all three femoral bone marrow subregions (femoral head, femoral neck, and greater trochanter), males showed a significantly higher PDFF than females (*p* ≤ 0.047). No significant differences in PDFF between males and females were observed in the two muscular compartments (gluteal and quadriceps muscles; *p* ≥ 0.707). Figure 3 displays representative colour-coded PDFF maps of the gluteal muscle (Fig. 3a, d), quadriceps muscle (Fig. 3b, e), and femoral neck compartments (Fig. 3c, f).

### Correlations between age, BMI, and PDFF of segmented compartments

**Fig. 4:**
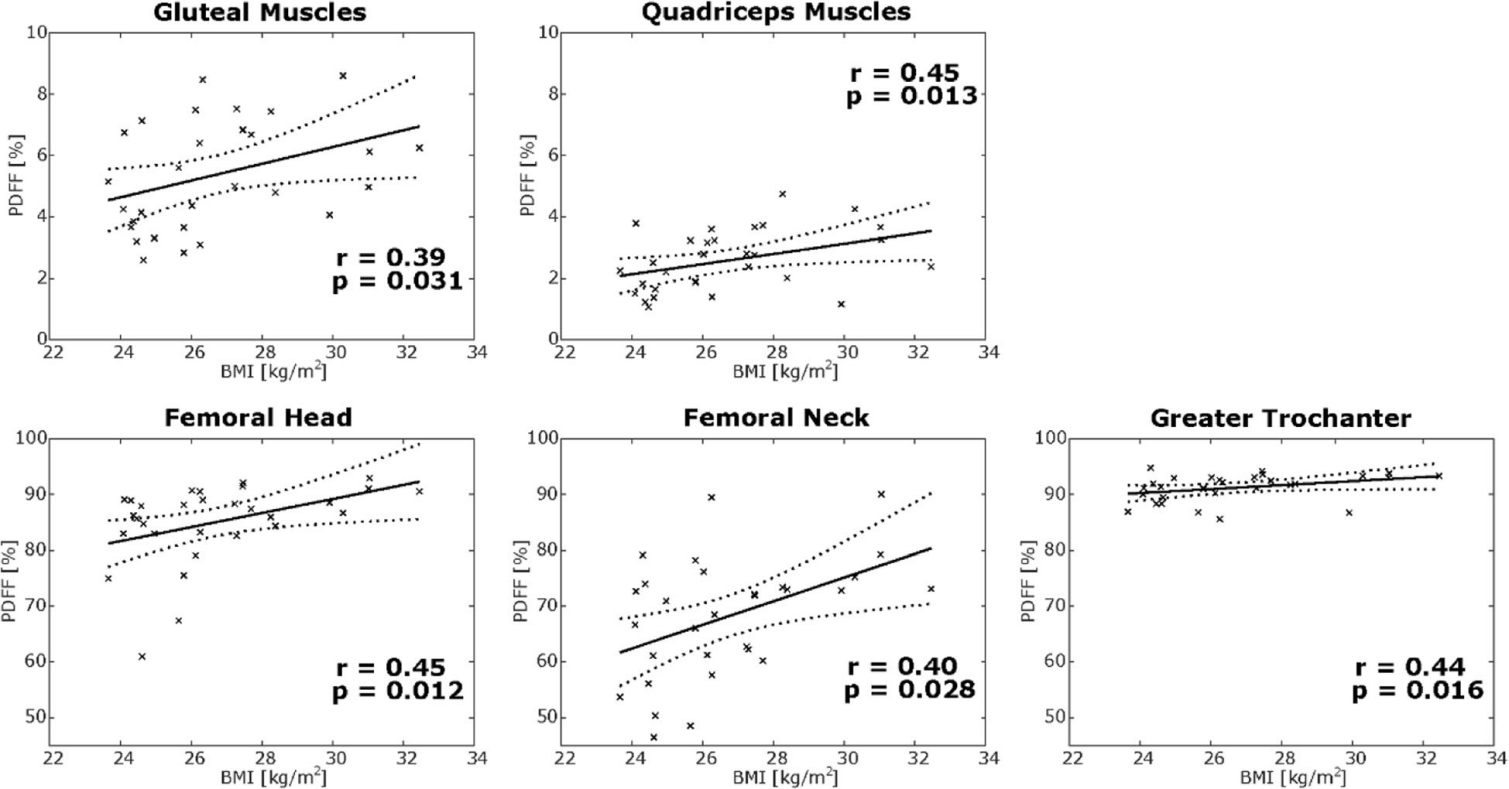
Scatter plots and linear fit (represented by the continuous line) of BMI *versus* PDFF of the two muscle compartments (*upper row*) and three femoral compartments (*bottom row*). The areas between the dotted lines represent the 95 % confidence intervals of the best linear fit. *BMI*, Body mass index; *PDFF*, Proton density fat fraction

There was no significant correlation between age and PDFF for any of the segmented compartments (*p* ≥ 0.225). The BMI showed only low but significant positive correlations with the PDFF of all segmented compartments (BMI *versus* PDFF_gluteus_: *r* = 0.394, *p* = 0.031; BMI *versus* PDFF_quadriceps_: *r* = 0.453, *p* = 0.013; BMI *versus* PDFF_femoral head_: *r* = 0.447, *p* = 0.012; BMI *versus* PDFF_femoral neck_: *r* = 0.402, *p* = 0.028; BMI *versus* PDFF_femoral greater trochanter_: *r* = 0.437, *p* = 0.016; Fig. 4).

Partial correlation analyses, controlled for age and BMI, revealed moderate but significant positive correlations (*p* ≤ 0.001) between the two muscular compartments (PDFF_gluteus_*versus* PDFF_quadriceps_) as well as between each of the femoral bone marrow subregions (PDFF_femoral __head_*versus* PDFF_femoral neck_, PDFF_femoral head_*versus* PDFF_femoral greater trochanter_, PDFF_femoral neck_*versus* PDFF_femoral greater trochanter_; Table 2). Regarding correlations between muscle and femoral bone marrow, only quadriceps and greater trochanter (PDFF_quadriceps_*versus* PDFF_femoral greater trochanter_) showed a significant correlation (*p* = 0.049; Table 2).

**Table 2:** Partial correlation analysis of proton density fat fraction of muscles and femoral subregions with age and body mass index as control variables

		Gluteus	Quadriceps	Femoral head	Femoral neck	Femoral greater trochanter
Gluteus	*R*	1	0.670	-0.184	-0.710	0.193
	*p*		< 0.001	0.348	0.720	0.325
Quadriceps	*R*		1	0.192	0.210	0.375
	*p*			0.328	0.283	0.049
Femoral head	*R*			1	0.655	0.613
	*p*				< 0.001	0.001
Femoral neck	*R*				1	0.638
	*p*					< 0.001
Femoral greater trochanter	*R*					1
	*p*					

### Reproducibility of measurements

The inter-reader reproducibility error of the PDFF was 0.27% (absolute units) and 7.22% (relative units) for the gluteal muscle groups, 0.09% and 5.71% for the quadriceps muscle groups, 1.56% and 2.33% for the femoral head, 1.07% and 1.81% for the femoral neck, and 1.24% and 1.39% for the greater trochanter bone marrow, respectively.

## Discussion

In the present study, no strong associations between muscle and femoral bone marrow PDFF were revealed at the proximal lower limb in young, healthy volunteers. However, moderate significant correlations of PDFF values were observed between the gluteal and quadriceps muscle groups (*r* = 0.670) and within the femoral bone marrow subregions (range: *r* = 0.613 to 0.655).

The study’s PDFF measurements of different muscle and bone marrow compartments of the thigh and hip region were performed in young men and women using CSE-MRI. PDFF values of the analysed proximal femur subregions (head, neck, and greater trochanter) were within the range of previously reported values [20]. Furthermore, male subjects exhibited significantly higher PDFF values than female subjects. This sex-dependent difference is in agreement with findings at the lumbar spine for a comparable age group [29, 30]. For all analysed compartments, the measured PDFF was positively associated with BMI but not with age, suggesting that in young subjects, fat deposition in the proximal lower limb musculature could be driven primarily by BMI rather than age. The excess accumulation of lipids in the human body characterising overweight and obesity, defined by an increased BMI, could be the primary reason for this finding.

Out of all analysed compartments, only one combination of muscular and femoral bone marrow compartments showed a borderline significant and rather weak correlation (quadriceps and greater trochanter, *r* = 0.375, *p* = 0.049). Thus, regarding PDFF, muscle and femoral bone marrow appear to be rather independent and there is no clear evidence for an interaction between skeletal muscle fat infiltration and bone marrow fat content, at least when it comes to a cohort of young, healthy subjects as investigated in this study. This may be in accordance with the previous finding of significant correlations at the lumbar spine between paraspinal muscles and vertebral bone marrow only amongst postmenopausal but not healthy premenopausal women [19]. Of note, the inter-reader reproducibility errors were good, amounting to values lower than 8% (absolute or relative units), respectively. This is on a similar scale like reported intra-reader reproducibility measurements for PDFF of 1.70% (absolute units) for vertebral bone marrow [31], 5.70% (relative units) for quadriceps muscles [32], and 0.05 to 0.72% (absolute units) for paraspinal muscle compartments [26], respectively.

Our study gained new insights into the physiology of muscle-bone interactions at the thigh and hip region. However, the following limitations have to be acknowledged. The relatively low number of participants and inclusion of only young, healthy subjects are limiting factors of the present work. The quadriceps femoris and gluteus muscle groups were both segmented as a whole, which can result in the inclusion of inter-muscular adipose tissue in the segmentations and should therefore be mentioned as a limitation of the present study. However, we decided to segment the entire muscle groups (*i.e*., quadriceps and gluteus) as they can be considered as functional units and for practical reasons because their individual components (gluteus maximus, gluteus medius, gluteus minimus and rectus femoris, vastus lateralis, vastus intermedius, and vastus medialis) cannot be clearly delineated in a considerable number of locations. Regarding the age dependence of the PDFF, one must keep in mind the limited age range of the study cohort. To further improve the understanding of muscle-bone interactions, in particular under pathophysiological conditions, future studies in older and diseased subjects, like osteoporotic or diabetic patients, are needed.

To summarise, the quadriceps and gluteal muscle compositions, assessed by CSE-MRI-based PDFF measurements at 3 T, were significantly associated with each other. Similarly, significant correlations of PDFF values were observed across the femoral bone marrow subregions. However, muscle and bone marrow compartments seem to be distinct from each other as there was no clear evidence for a strong interaction between muscle fat infiltration and bone marrow fat content at the thigh and hip region of healthy young subjects. Given the preliminary character of this study, further investigations may confirm or disconfirm the results in larger cohorts of healthy subjects as well as in patients that show disease-related muscle and/or bone marrow alterations

## Data Availability

The datasets used and/or analysed during the current study are available from the corresponding author on reasonable request.

## Abbreviations

BMI: Body mass index
CSE-MRI: Chemical shift encoding-based water-fat magnetic resonance imaging
IQR: Interquartile range
MRI: Magnetic resonance imaging
PDFF: Proton density fat fraction
SD: Standard deviation
